# Oxidative stress and life histories: unresolved issues and current needs

**DOI:** 10.1002/ece3.1790

**Published:** 2015-11-17

**Authors:** John R. Speakman, Jonathan D. Blount, Anne M. Bronikowski, Rochelle Buffenstein, Caroline Isaksson, Tom B. L. Kirkwood, Pat Monaghan, Susan E. Ozanne, Michaël Beaulieu, Michael Briga, Sarah K. Carr, Louise L. Christensen, Helena M. Cochemé, Dominic L. Cram, Ben Dantzer, Jim M. Harper, Diana Jurk, Annette King, Jose C. Noguera, Karine Salin, Elin Sild, Mirre J. P. Simons, Shona Smith, Antoine Stier, Michael Tobler, Emma Vitikainen, Malcolm Peaker, Colin Selman

**Affiliations:** ^1^ Institute of Biological and Environmental SciencesUniversity of AberdeenTillydrone AvenueAberdeenAB24 2TZUK; ^2^ State Key Laboratory of Molecular Developmental BiologyInstitute of Genetics and Developmental BiologyChinese Academy of SciencesBeijingChina; ^3^ Centre for Ecology and ConservationUniversity of ExeterPenryn CampusCornwallTR10 9FEUK; ^4^ Department of Ecology, Evolution and Organismal BiologyIowa State University251 Bessey HallAmesIowa50011; ^5^ Physiology, Barshop Institute for Aging and Longevity ResearchUTHSCSA15355 Lambda DriveSan AntonioTexas78245; ^6^ Department of BiologyLund UniversitySolvegatan 37Lund223 62Sweden; ^7^ The Newcastle University Institute for AgeingInstitute for Cell & Molecular BiosciencesCampus for Ageing and VitalityNewcastle upon TyneNE4 5PLUK; ^8^ Institute of Biodiversity, Animal Health and Comparative MedicineUniversity of GlasgowGraham Kerr BuildingGlasgowG12 8QQUK; ^9^ University of Cambridge Metabolic Research Laboratories and MRC Metabolic Diseases Unit, Level 4Wellcome Trust‐MRC Institute of Metabolic ScienceAddenbrooke's HospitalCambridgeCB2 0QQUK; ^10^ Zoological Institute and MuseumUniversity of GreifswaldJohann‐Sebastian Bach Str. 11/12Greifswald17489Germany; ^11^ Behavioral BiologyUniversity of GroningenNijenborgh 7Groningen9747 AGThe Netherlands; ^12^ MRC Clinical Sciences CentreImperial College LondonHammersmith Hospital CampusDu Cane RoadLondonW12 0NNUK; ^13^ Department of ZoologyUniversity of CambridgeCambridgeCB2 3EJUK; ^14^ Department of PsychologyUniversity of MichiganAnn ArborMichigan48109; ^15^ Department of Biological SciencesSam Houston State University1900 Avenue ILDB 100BHuntsvilleTexas77341; ^16^ Department of Animal and Plant SciencesUniversity of SheffieldAlfred Denny Building, Western BankSheffieldS10 2TNUK; ^17^ Department Ecology, Physiology et EthologyUniversity of Strasbourg ‐ IPHC (UMR7178)23, rue BecquerelStrasbourg67087France; ^18^ Rushmere13 Upper CroftsAllowayKA7 4QXUK

**Keywords:** Aging, disposable soma theory, free radicals, life‐history theory, oxidative stress

## Abstract

Life‐history theory concerns the trade‐offs that mold the patterns of investment by animals between reproduction, growth, and survival. It is widely recognized that physiology plays a role in the mediation of life‐history trade‐offs, but the details remain obscure. As life‐history theory concerns aspects of investment in the soma that influence survival, understanding the physiological basis of life histories is related, but not identical, to understanding the process of aging. One idea from the field of aging that has gained considerable traction in the area of life histories is that life‐history trade‐offs may be mediated by free radical production and oxidative stress. We outline here developments in this field and summarize a number of important unresolved issues that may guide future research efforts. The issues are as follows. First, different tissues and macromolecular targets of oxidative stress respond differently during reproduction. The functional significance of these changes, however, remains uncertain. Consequently there is a need for studies that link oxidative stress measurements to functional outcomes, such as survival. Second, measurements of oxidative stress are often highly invasive or terminal. Terminal studies of oxidative stress in wild animals, where detailed life‐history information is available, cannot generally be performed without compromising the aims of the studies that generated the life‐history data. There is a need therefore for novel non‐invasive measurements of multi‐tissue oxidative stress. Third, laboratory studies provide unrivaled opportunities for experimental manipulation but may fail to expose the physiology underpinning life‐history effects, because of the benign laboratory environment. Fourth, the idea that oxidative stress might underlie life‐history trade‐offs does not make specific enough predictions that are amenable to testing. Moreover, there is a paucity of good alternative theoretical models on which contrasting predictions might be based. Fifth, there is an enormous diversity of life‐history variation to test the idea that oxidative stress may be a key mediator. So far we have only scratched the surface. Broadening the scope may reveal new strategies linked to the processes of oxidative damage and repair. Finally, understanding the trade‐offs in life histories and understanding the process of aging are related but not identical questions. Scientists inhabiting these two spheres of activity seldom collide, yet they have much to learn from each other.

## Background

First formulated in the 1950s (Harman 1956), the free radical damage theory of aging enjoyed a golden period as the predominant mechanistic theory of aging (Beckman and Ames 1998). In the new millennium, however, its luster has been somewhat tarnished, with a series of studies providing data contrary to its main theoretical expectations [e.g., (Yang et al. 2007; Doonan et al. 2008; Jang et al. 2009; Perez et al. 2009c; Zhang et al. 2009)]. These findings have led to a number of review articles pronouncing the theory dead, moribund, or at the very least suffering a mid‐life crisis (Buffenstein et al. 2008; Gems and Doonan 2009; Perez et al. 2009a; Speakman and Selman 2011; Stuart et al. 2014 but see Barja 2013ba). Many gerontologists now question the idea that oxidative stress is the principal, generalized mechanism underlying aging, although most agree it probably plays some role. On the other hand, eco‐physiologists have embraced this concept as providing a physiological mechanism that might play a key role in determining the outcome of life‐history trade‐offs between activities, such as growth or reproduction, and body maintenance and hence future survival or reproduction (Costantini 2008; Dowling and Simmons 2009; Metcalfe and Alonso‐Alvarez 2010; Isaksson et al. 2011a,b). Over the last decade, there has been a proliferation of studies investigating whether oxidative stress plays a role in life‐history evolution. Such investigations have been largely correlative, but sometimes experimental, and undertaken across a diversity of organisms, studied under natural, semi‐natural, and laboratory conditions (summarized and subjected to meta‐analysis in Blount et al. (2015)). The results, however, have often been contradictory. The current absence of consensus and direction has led to the need to crystallize the main issues in the field, to avoid the area drifting into a shambles of conflicting data, and to resolve contradictory claims about what the data actually mean. This manuscript emerged from a workshop attended by the authors and held in April 2014, funded by the Rank Prize Organization. It reflects a consensus on the main unresolved issues in this field, as perceived by the participants of the workshop, and the theoretical and empirical approaches required to resolve them. Our hope is that this commentary will help to move the field forward positively by stimulating researchers to refine and expand the scope of existing studies so that we do not simply continue to accumulate the same types of data, resulting in repeated additions to the confusion, as opposed to genuine illumination. In a wider context, we hope that by focussing the attention of the wide variety of researchers in this area it may additionally rejuvenate the interests that gerontologists have in oxidative stress – who were perhaps too hasty to declare the idea deceased (Kirkwood and Kowald 2012). We identify 6 Themes that are not mutually exclusive and are not listed in any perceived order of importance. These are (1) to understand the significance of different responses to reproduction observed in different tissues, (2) to refine the methodology for measuring oxidative stress to allow studies in the field that do not require precious animals in longitudinal studies of life history to be sacrificed, (3) to make laboratory studies more representative of conditions in the wild, (4) to develop good theoretical models enabling refinement of predictions of what we expect experiments to show, and the development of good alternative ideas, (5) to expand the range of species and processes that are studied to gain new perspectives and insights, and (6) to foster greater communication between evolutionary/physiological ecologists and biogerontologists. We stress that it is entirely possible for the management of oxidative stress to play a role in life‐history evolution and that the need to manage, minimize, or repair oxidative damage might vary among different kinds of tissues and organisms. That such management occurs might well mean that oxidative stress, at least in simple terms of accumulated damage to macromolecules, also has a role in the aging process.

### Theme 1: variable responses of different tissues and different macromolecular targets during reproduction

As data have accumulated, it has become clear that different studies have utilized an extensive range of assays to directly (and indirectly) quantify oxidative stress in different tissues. Much has been written about the need to include direct measures of oxidative damage and/or reactive oxygen species (ROS), rather than only measure antioxidants as a proxy measure, as it is the accumulated damage that is presumed to be harmful (Monaghan et al. 2009; Isaksson et al. 2011a,b; Selman et al. 2012; Metcalfe and Monaghan 2013). In response, a growing number of studies have measured markers of oxidative damage in relation to reproduction. Much of this research has focussed on mammals, mostly rodents, and almost exclusively on females. While some mammalian studies have supported the idea that investment in offspring production (during pregnancy and lactation) causes oxidative damage (Sainz et al. 2000; Bergeron et al. 2011; Stier et al. 2012; Fletcher et al. 2013), others have found no effect or that oxidative damage is actually reduced in lactating compared to nonreproducing females (Garratt et al. 2011, 2013; Oldakowski et al. 2012; Schmidt et al. 2014). The biological relevance of this diversity in response is unclear and has often been explained by individual, species, tissue or assay differences, and the difference between studies in the laboratory and field (see Themes 2 and 3 below). However, it has recently become apparent, by applying multiple assays across multiple tissues, that it is possible, in exactly the same individuals, to find some tissues and macromolecules where reproduction elevates damage, others where there is no impact, and yet others where damage is reduced (Garratt et al. 2011, 2013; Oldakowski et al. 2012; Yang et al. 2013). These complex patterns may result from individuals selecting which tissues to protect and which to leave vulnerable (Garratt et al. 2013; Yang et al. 2013). An intriguing example of this complexity is the lack of an effective antioxidant system in mammalian pancreatic beta‐cells, which renders them susceptible to oxidative stress. Rashidi et al. (Rashidi et al. 2009) have shown how this phenomenon can be explained in terms of life‐history trade‐offs whereby reactive oxygen species (ROS) in beta‐cells, by their negative effect on insulin synthesis/secretion, play a fitness‐enhancing role for the whole organism.

One key problem is that we do not know what the long‐term consequences (if any) of different forms of oxidative damage are, for both organ function and ultimately survival, and whether changes in oxidative damage during reproduction are reversible/repairable. Furthermore, we do not know whether the consequences of oxidative damage differ across the life course. An automobile analogy may help conceptualize this problem. In the course of being driven, cars get dirty, and it is easy to imagine that oxidative damage is akin to cellular ‘dirt’. Where exactly the dirt lands on a car may have very different consequences. For example, dirt on the body work may be unsightly, and easy to quantify, but it has little impact on the functionality of the car. Dirt on the windscreen/windshield may be more serious, and impair the performance of someone driving the car. But dirt in the fuel line or carburetor could completely prevent the car from operating. At present, we do not know which parts of an animal, that become oxidatively damaged during reproduction, or throughout life, are analogous to the body work (which can be restored to pristine form if washed and waxed), and which are the fuel lines and carburetor. There is a strong need therefore for studies that associate different measurements of damage with functional outcomes such as organ function and fitness outcomes such as survival and future reproductive performance.

We do not wish to completely discourage people from making studies where single tissues are sampled, and single markers of damage and protection are employed; in studies of natural populations of animals, this is sometimes the only possible sampling strategy (Selman et al. 2012) (see also Theme two). However, it is important to recognize that the correlation of responses across different tissues in the same individual can be poor, at least based on the relatively small number of studies that have explored such associations during reproduction (Yang et al. 2013; Schmidt et al. 2014; Xu et al. 2014) or in response to other stressors (Selman et al. 2002a,b, 2008; Kaushik and Kaur 2003; Veskoukis et al. 2009; Meitern et al. 2013). Hence, interpreting such studies in the absence of a clear link to a functional outcome is fraught with difficulty. In addition, we sorely need more studies that expand the range of markers that are used and the range of tissues that are measured, in response to experimental manipulations of both reproductive status (allocated to breed or not breed) and reproductive effort (number of raised offspring or investment per offspring). It is important when making such manipulations to quantify the impact of the manipulation on the investment by the female, as simply adding or subtracting offspring does not necessarily cause an impact on female investment (Speakman and Garratt 2014). This is because individuals may continue to work at the same rate they would have without the manipulation, generally with the outcome of the manipulation being felt more acutely on the offspring than the adult. At present, we have no idea whether conservation exists across different organisms when examining multi‐tissue and multi‐target patterns of damage and protection. Presumably, organisms allocate protection and repair to those things that are most critical, which may itself vary with life stage. Hence, patterns of protection and repair may provide useful clues as to the targets most likely to be functionally significant for survival. At present, we do not know whether the same targets will be universally important across all organisms, or whether different taxa will have unique points of vulnerability. Moreover, it is unclear whether the effects will be the same across both sexes, in particular as males and females may show pronounced differences in markers of oxidative stress during reproduction (Alonso‐Alvarez et al. 2004; Wiersma et al. 2004; Isaksson et al. 2011a,b; Stier et al. 2012; Isaksson 2013), and in their rates of aging. Resolving these issues will be an important future aim as our knowledge expands.

### Theme two: the dilemma of field studies. Longitudinal data on life histories make animals too valuable to sample destructively for oxidative stress assays

Linking together information on oxidative stress and life‐history strategies across the lifespan requires detailed knowledge of individuals at different ages/stages of their life histories. Longitudinal studies and experimental manipulations of either status or effort provide the best way to understand the nature of the underlying associations because cross‐sectional surveys and correlational studies may be prone to artifacts through differential survival and reproduction (Nussey et al. 2009; Bouwhuis et al. 2012). This might, for example, lead to the illusion that oxidative damage declines in relation to organismal age, because those individuals with the highest levels of damage die sooner. Additionally, this could suggest oxidative damage is reduced by reproduction, because only those individuals with pre‐existing low damage initiate attempts to reproduce [e.g., Beaulieu et al. 2014]. Field studies that meticulously follow individuals throughout their life course, quantifying reproduction and growth, are growing in number. Repeatedly measuring oxidative stress markers within individuals over time seems an obvious way to test the role of oxidative stress on life histories (Nussey et al. 2009; van de Crommenacker et al. 2011). There is, however, a major dilemma because in general such studies were not initiated as tests of the oxidative stress theory, and they have alternative goals and aims that may be conflicting. For example, the currently available assays, apart from measurements in blood and minor biopsies, require destructive testing. Euthanizing animals that are part of long‐term monitored populations is, however, generally incompatible with the primary goals of such longitudinal field studies. This might not be a problem if it turns out that the markers measured in blood are indeed the markers of greatest functional significance (see Theme 1 above). This would be indeed fortuitous. However, if these markers only reflect “dirt on the chassis” then the opportunity to exploit the thousands of person hours devoted to longitudinal quantification of the life‐history parameters of known individuals will be lost. There is a major demand therefore for the development of assays in accessible samples that will inform us about the multi‐tissue, multi‐target nature of protection and repair processes without the need to kill (or severely impair) animals in the process. For example, standard tissue biopsies may be sufficiently traumatic to animals that even though the animal survives the tissue collection, it may have a significantly elevated risk of mortality when placed back into the field, such that its contribution to the population demographic data may be compromised. Alternatively, its behavior might be altered leading to reduced likelihood of it being resampled at a later age. Moreover, tracking, trapping, capturing, and restraining wild animals may affect measures of oxidative status. Nevertheless, some tissues (e.g., feathers, hair, and small epidermal biopsies) may be readily sampled repeatedly with minimal invasiveness, and considerable information can also be gleaned from urine and fecal samples. We envisage that technological advances in the future may come in several different forms. First, it is already clear that compounds in the urine/feces may provide an indication of oxidative damage (e.g., levels of f2 isoprostanes, 8‐OHdG). As metabolite profiling of urine/feces becomes more sophisticated with the capacity to monitor simultaneously thousands of compounds, the prospect that multiple urinary markers that may provide a “damage fingerprint” seems feasible. Second, animals may be injected with compounds that are metabolized in relation to aspects of “damage” or “protection/repair” capacity and the products of such reactions in blood or urine monitored. It may be possible to use radioactive markers to localize tissue damage or protection in given tissues using PET scanning or other imaging technology – although admittedly applying such technology in the field would prove challenging. Finally, as the costs of transcriptomic profiling by RNAseq decline, it is now possible to generate profiles of gene expression from nucleated blood cells, mouth swabs and/or very small biopsy samples, allowing simultaneous evaluation of the status of repair, turnover, and protection systems, in multiple tissues. Development of such methods would provide significant benefits for both field‐ and laboratory‐based studies. A major point, however, that we wish to emphasize is that it is not a trivial matter to bring assays from the laboratory to the field, and needs to be done with great care. Assays may be highly sensitive to environmental conditions and individual factors that while easy to control in the laboratory are much more problematical to control in the field – including for example time as last meal (or the composition of the meal itself), whether or not the individual encountered a predator or a dominant individual in the recent past, and the almost unavoidable influence of pollutants in all but the most pristine environments. Just because a particular assay works well under controlled laboratory settings, we should not assume that it will work equally well in the field.

### Theme three: considerations for laboratory studies

Laboratory studies provide unrivaled opportunities to perform controlled experiments, with multiple detailed measurements and terminal assays that are seldom possible in the field. Yet these studies come with their own drawbacks, chief among which is the question whether the situation in the laboratory adequately mimics the situation in the field with respect to the key elements that generate the trade‐off between reproduction and survival. An important question is whether animals in the laboratory with *ad libitum* food supplies are in a situation where it is necessary to trade‐off somatic protection against reproduction (e.g., Selman et al. 2012; Metcalfe and Monaghan 2013). This could be a serious issue if animals in the field are routinely food limited. However, if animals in the field during peak reproduction are operating at an intrinsically determined physiological capacity, then this issue is less important because both laboratory and field animals will be likely to be working at this same limiting physiological capacity – that is the energy budget within which different costly functions must be accommodated will be the same irrespective of food supply (Speakman and Garratt 2014). There is considerable evidence to suggest that some strains of laboratory mice and other rodents are operating at a physiologically imposed maximum when they are at peak lactation (reviewed in Speakman and Krol 2011). However, the nature of this ceiling is disputed. Similarly in the field, there is evidence that some species of bird are operating at a physiological ceiling (Daan et al. 1996; Tinbergen and Verhulst 2000; Welcker et al. 2010; Elliott et al. 2014), although the causes and definition of this ceiling are also unclear. How commonly physiological factors limit expenditure, as opposed to the level of food supply is uncertain, but studies of the responses of animals to food supplementation indicate that the response is seldom to elevate energy expenditure (Boutin 1990), instead opting to reduce the time required to ingest a given level of resource, perhaps indicating that physiological limitations may be the norm.

Nevertheless, there are other aspects of the laboratory situation that could compromise experiments to establish the oxidative costs of reproduction, and or the implications of oxidative damage for survival. Among such issues are the levels of antioxidants in laboratory rodent chow, when compared with wild foods (assuming dietary antioxidants do play a role in damage mitigation – which is currently unclear). This could be a problem in different ways. Manufacturers of rodent chow generally add large amounts of antioxidants, not as a nutritional additive but to improve the shelf life of the product. Such levels may be unnaturally high relative to foods available in the wild. As food intake is greatly elevated during lactation, it is possible that reproducing laboratory animals may inadvertently ingest enormous levels of exogenous antioxidants in their diets, and this may explain why a common observation in laboratory studies is that oxidative damage at peak lactation appears to be reduced. Should animals studied in a laboratory context therefore be fed diets with extremely low levels of antioxidants? The problem is that we do not presently know the extent to which lactating mammals, or chick‐rearing birds in the wild, modulate their dietary intake to enhance their antioxidant intake or intake fatty acids and other nutritional components that are less susceptible to attack by free radicals (Beaulieu and Schaefer 2013). There is a need for information on the dietary intakes of antioxidants of the diet in wild animals at critical times of peak reproductive activity so that diets could be formulated to allow laboratory studies to more closely mimic the situation in the wild [see also Isaksson et al. (2011a,b)].

Another issue relates to the ambient temperatures and photoperiods at which laboratory animals are maintained. In the wild, animals are exposed to fluctuating temperatures and photoperiods which place them under variable thermoregulatory demands (endotherms) or constraints (ectotherms). This might compromise the ability to invest in reproduction [e.g., Krol and Speakman (2003)]. Yet in the laboratory, studies are commonly performed at room temperature (c. 21–23°C) and a fixed photoperiod with minimal fluctuations being part of the legal housing requirements in many countries. The stability of this temperature and its level and invariant photoperiod may be completely inappropriate to mimic the experience of animals in the wild. Levels of many hormones are driven by photoperiod changes, and these often prepare the organism for the change in reproductive status altering not only the hypothalamic–pituitary gonad axis but also metabolic rate, membrane composition appetite, and stress responses. Moreover, melatonin, which directly depends on photoperiod, and has powerful antioxidant properties, can affect the overall oxidative status of the organism (Tan et al. 2010). For some animals which routinely occupy hypoxic environments in the wild, for example, fossorial animals or those from high altitude, well‐ventilated facilities may actually expose them to unnaturally high levels of oxygen and hence risk of oxidative stress. A classic example in this regard are the high levels of oxidative damage in naked mole rats (*Heterocephalus glaber*) (Andziak and Buffenstein 2006; Andziak et al. 2006), which is an exceptionally long‐lived rodent. Naked mole rats naturally live in large family groups in sealed underground burrows where they experience profound hypoxia and hypercapnia (Peterson et al. 2012). In the laboratory, high levels of oxidative stress may be a result of the relatively hyperoxic captive conditions. The fact naked mole‐rats in captivity tolerate this chronic high level of oxidative damage and their extraordinary longevity is based upon captive data (Lewis et al. 2013) may suggest oxidative stress is not a key element of their enhanced longevity. An under‐recognized issue is also the possibility that laboratory animals live continually in a state of chronic stress, even in the best‐managed facilities. Even such apparently subtle changes such as rotation of staff caring for the animals [e.g., Sorge et al. 2014] may influence the stress experienced by captive animals.

Finally, if the intention is to establish the linkage between aspects of damage and survival, it is important to recognize that oxidative stress may only be a survival issue under certain environmental conditions. For example, if animals sustain damage to their skeletal muscle, this might only compromise individuals if they have to perform physical exercise to obtain their food or avoid predation – as is generally the case in the wild, but seldom so in captivity. Another example is if oxidative stress acts to reduce the functionality of the immune system. However, for animals raised in specific pathogen‐free (SPF) facilities such an effect would be unlikely to translate into a survival impact.

### Theme four: the lack of specificity in the theoretical predictions or of good alternative hypotheses

The idea that the link between reproduction and future survival may be mediated by oxidative stress predicts that oxidative damage should increase as a consequence of reproduction and that this should limit future survival or future reproductive performance. These predictions are not the same as the predictions surrounding the role of oxidative stress as a mediator of aging. Hence, tests of the free radical theory of aging are not necessarily equivalent to tests of the mediating role of oxidative damage in life histories. Moreover, a problem with this prediction is that it is completely nonspecific regarding the locations and targets of such damage. The organism is viewed as a single integrated and homogeneous unit that is predicted to be damaged or not damaged. In reality, organisms comprise a complex set of organs that perform very different functions, have different metabolic rates, different levels of oxidants and antioxidants, repair mechanisms and ROS, and these organs are potentially affected very differently by the process of reproduction. For example, for mammals and birds, a major consequence of reproduction is elevated food intake. This food needs to be digested by the alimentary tract and the digested substrates then processed by the liver. We might imagine major impacts of reproduction on the metabolic activities of these tissues. Indeed in mice, at peak lactation the liver doubles in size and the small intestine grows longer by around 50% (Johnson et al. 2001). In contrast, the brain needs to function continuously whether the organism reproduces or not. It seems unlikely that major changes occur in brain metabolic rate during reproduction, and its size is unaffected. These changes in size and potentially also the metabolic rate of the component body tissues during reproduction may be coupled with alterations in where the animals decide to selectively allocate their protection and repair processes. However, we do not have any clear predictions regarding what the consequences of these differences might be. Hence, it is difficult to evaluate the data that are being generated. Does the theory predict that damage should be uniformly elevated across all tissues? If so we probably have enough data to reject it already. However, perhaps the model does not predict this. For example, the proliferation of newly constructed, and presumably undamaged, liver tissue during reproduction may mean that the average concentration of measured damage is reduced – and this would be consistent with the empirical observations in several previous studies (Garratt et al. 2011, 2013; Oldakowski et al. 2012; Yang et al. 2013; Xu et al. 2014). In fact, many organs in lactation show hyperplasia and this may explain reduced damage reported more widely in other tissues such as the kidney (Oldakowski et al. 2012; da Silva et al. 2013). Hence, these reduced levels of damage may not be inconsistent with the life‐history predictions, despite on the face of it being in the completely opposite direction to the simplistic prediction that “damage will be increased”. Furthermore, even within the cell, there may be nonuniformity in the distribution of sites at which oxidative damage occurs, is detected by conventional assays, and actually matters. There is a strong need therefore for a more refined conceptualization of the idea, and derivation of testable mathematical models which make predictions at the tissue/organ level, at a particular stage of development and with respect to different macromolecular targets of damage. We envisage these models may generate different predictions depending on the actual mechanics of reproduction. For example, pregnancy and lactation might be expected to involve very different physiological processes and consequences, than oviparity and chick feeding in birds. Viviparous and oviparous reptiles may similarly differ in the patterns of oxidative stress that are predicted by the theory.

A second element of the current theoretical shortcomings is that not only are the predictions of the oxidative stress model for life‐history effects poorly defined, but there are few alternative hypotheses to be evaluated. Even the changes that are anticipated under the null hypothesis remain uncertain, as for example the changes in tissue proliferation during reproduction highlighted above might be expected to alter apparent measured damage, even if there was no actual change in the underlying processes. Hence, the required modeling needs not only to refine the predictions of the oxidative stress model but also the expectations under the null and alternative hypotheses. One promising potential alternative is the “oxidative shielding theory” (Blount et al. 2015). This idea suggests that there may be major consequences of oxidative damage early in life. Hence, a priority of the parents may be to ensure that damage to their soma(s) is not transferred to their offspring. Even though oxidative protection and repair may be costly, it may be more beneficial for parents to upregulate these during reproduction to reduce their own oxidative damage, thereby minimizing damage to their offspring, because the fitness benefits in offspring quality, offset the sacrifice in terms of offspring numbers. Clearly, transfer of somatic damage from parents to offspring may be more likely during some aspects of reproduction (e.g., in pregnancy where the fetus and mother are in close physiological contract and the mother passes chemicals from her body to the fetus) compared with others like chick rearing in birds where there is no direct physiological contact of parents and offspring. This may suggest shift in focus toward egg production and laying (Vezina and Williams 2002; Williams et al. 2009) might be warranted (see also Velando et al. 2008).

### Theme five: a wider diversity of animals and experimental conditions on which to test the ideas may provide useful insights

Tests of the oxidative stress theory for the trade‐off in life‐history evolution have been performed on a wide variety of vertebrate species including small mammals (Bergeron et al. 2011; Garratt et al. 2011; Fletcher et al. 2013; Cram et al. 2015), including bats (Wilhelm et al. 2007; Schneeberger et al. 2014), large mammals (Castillo et al. 2005; Nussey et al. 2009; Rizzo et al. 2013), reptiles (Robert et al. 2007; Isaksson et al. 2011a,b), and birds (Wiersma et al. 2004; Costantini et al. 2006, 2010; Bize et al. 2008; Marko et al. 2011; Isaksson 2013). Yet, despite this large range, there are a wide variety of unstudied reproductive strategies within the endotherms, including semelparity, and brood parasites where contrasting behaviors of the participants may make useful tests of the model. Moreover, within ectotherms, the growing number of long‐term mark/recapture studies (e.g., Robert and Bronikowski 2010; Schwanz et al. 2011) should allow a more complete understanding of oxidative stress and damage with respect to physiological mode and temperature sensitivity of the animals. Moreover, future analyses may enable partition of the contrasting results between mammals and birds into causes due to phylogeny and physiology (reviewed in Schwanz et al. 2011).

Expanding the diversity of models used may provide unexpected insights into the processes involved that are not immediately obvious from the limited set of animals studied thus far. Insights from the long‐lived social breeding naked mole rat, for example, have been particularly important regarding the complexity of the process of damage and protection, as the extant levels of oxidative damage are surprisingly high for such a long‐lived organism (Andziak et al. 2006): if the oxidative stress theory of aging is correct. Moreover, contrasting the role of oxidative stress in life histories, levels of damage are similar among breeding females and nonbreeding subordinates, despite a 3–5 fold change in metabolic rate during pregnancy and lactation (Urison and Buffenstein 1995). High levels of oxidative damage even in young animals have also been observed in long‐lived bats and birds (Hamilton et al. 2001; Hermes‐Lima and Zenteno‐Savin 2002; Buffenstein et al. 2008) and suggests an understudied strategy for coping with oxidative stress may just be to tolerate the damage by mitigating its functional impact (Lewis et al. 2013) (see Theme 1). Tolerance of damage may be attributed to subcellular localization of the oxidatively damaged macromolecules, a phenomenon seldom measured in studies of oxidative damage and life history. Naked mole‐rats, in particular, have an enhanced network of mechanisms to maintain protein stability when challenged with oxidative stressors, as indicated by findings that their liver proteins are significantly more resistant to urea‐induced protein damage (Perez et al. 2009b). Moreover, it appears that certain proteins bear the brunt of the oxidative damage (e.g., triosephosphate isomerase and peroxiredoxin 1), yet their functionality does not appear to be compromised (De Waal et al. 2013). These proteins may serve as oxidative sinks, which lead to better protection of the cytosolic environment from the formation of harmful oligomers or larger aggregates (Rodriguez et al. 2014).

Although different species have different life‐history strategies and these life‐history differences may engender different responses to oxidative stress, comparisons across species are accompanied by a suite of still‐debated difficulties, such as the problems of how to deal with body size effects and phylogenetic independence (Speakman 2005; Chamberlain et al. 2012; Barja 2013bb). Studying the co‐variation of life‐history strategies and oxidative damage within single species avoids the allometry and scaling issues that arise when making comparisons across species. For example, contrasts between “fast” and “slow” living garter snakes have been particularly informative with respect to the evolution of physiological divergence including patterns of oxidative and other stressors (Schwartz and Bronikowski 2011, 2013a,b). Their contrasting reproductive strategies and stress responses could expand our understanding of the universality of oxidative stress as mediator of life histories, particularly interpreted within a coherent theoretical framework (Theme 4) that generates contrasting predictions of what we might expect to happen. Overall, we see distinct advantages to broadening the scope of existing studies to include a wider diversity of organisms exploiting a more expansive diversity of life‐history strategies.

### Theme six: evolutionary/physiological ecologists and gerontologists need to speak to each other more deeply and more often

The idea that oxidative stress might underpin the phenomenon of aging was the dominant mechanistic theory of aging for almost 50 years. Although the idea that oxidative stress may play a role in life‐history trade‐offs is not the same, it is clear that the two are related, as reproductive lifespan, reproductive senescence, and mortality risks, hence lifespan, are all components of life histories. However, these theories do make different predictions, and testing them requires different approaches. The popularity of the “free radical oxidative damage” theory of aging among gerontologists waned, almost in parallel with the increase in popularity of the role of oxidative stress in influencing the outcome of life‐history trade‐offs among eco‐physiologists. It is apparent from the literature cited in many ecophysiology texts, which frequently include the original paper on free radicals by Harman (1956) and even the rate of living theory by Pearl (Pearl 1928), that many of the more recent contributions to the literature by biogerontologists, which have fueled the reduction in the popularity of the idea in that field, are either not being read, or are being selectively ignored. Examples include the impressive body of work by Arlan Richardson and colleagues showing that knocking out or over expressing many of the major protective enzymes against oxidative stress, such as superoxide dismutase, catalase and glutathione peroxidase, leads to the expected changes in oxidative damage, but with no impact on lifespan (Perez et al. 2008, 2009c; Jang et al. 2009; Zhang et al. 2009) (but recall discussion above under Theme 3 regarding the problems of laboratory studies). However, when these genetically manipulated models are subjected to high‐fat diets or other unfavorable environmental conditions, increased oxidative stress accelerates pathology and shortens lifespan, and reduced oxidative stress and overexpression of antioxidants reduces age‐associated pathology and increases lifespan (Salmon et al. 2010). These examples clearly suggest that the role that oxidative stress plays in aging depends on environmental conditions. Under optimized laboratory, husbandry conditions animals can tolerate oxidative damage. However, under environmental conditions including suboptimal nutrition, oxidative damage, in keeping with the oxidative stress theory of aging, may overwhelm the cytoprotective defences with concomitant effects on age‐related diseases and lifespan (Salmon et al. 2010).

Moreover, many studies by both evolutionary/physiological ecologists and biogerontologists still reiterate the idea that increases in energy expenditure lead to increases in free radical production, despite the evidence in favor of that being at best equivocal. Papers predicting, on the basis of our improved knowledge of mitochondrial functioning, that the opposite is likely to pertain in many circumstances were published 15 years ago (Brand 2000) and empirical studies showing that high metabolism is linked to greater longevity or is unrelated to lifespan, in both correlational and experimental settings, have been published also within the last decade (Speakman 2004; Selman et al. 2008; Keipert et al. 2011). It seems that the precise mitochondrial pathway responsible for the increased energy expenditure (*i.e.,* mitochondrial proton leak *vs*. ATP synthesis) is likely to determine the sign of the relationship between energy expenditure and oxidative stress levels (Stier et al. 2014). This selective blindness to contrary evidence occurs despite the fact that several reviews of the field (e.g., Monaghan et al. 2009; Selman et al. 2012; Speakman and Garratt 2014) have pointed out the problem with the assumption of a direct positive link in considerable detail.

Recent findings in gerontology that have emphasized the roles of particular signaling pathways in the process of aging and senescence (such as the insulin/IGF‐1 signaling, sirtuin, and mTOR pathways), which play highly conserved roles in growth metabolism and reproduction (Kenyon 2011; Gems and Partridge 2013), and the potential role of multiplex resistance to stressors based on responses of cultured primary fibroblasts (Salmon et al. 2005; Harper et al. 2007, 2011) have much to offer ecophysiologists as they strive to understand the physiological basis of life‐history trade‐offs. In addition, many biobiogerontology laboratories have access to equipment for quantifying various aspects of stress that are beyond the reach of a dedicated ecophysiology laboratory, and hence, collaborations between the two fields may be extremely useful. But the flow of information and technology need not be only in one direction. Furthermore, it is now becoming clearer that ROS themselves participate in signaling pathways. In addition to the long‐recognized role of ROS in host defences against pathogens, ROS have also been shown to be an important feature in the regulation of the entry of mammalian cells to the state of replicative senescence. The senescent state of the cell is not merely the result of intrinsic failure of the mitotic machinery, but appears to be a highly regulated outcome that results from activation of a pathway that integrates input from mitochondrial dysfunction, telomere erosion, and DNA damage (Passos et al. 2010).

Biogerontologists may have been too hasty to dismiss the role of oxidative stress as an important functional component of aging, based on studies of model organisms in protected laboratory environments (Theme 3). Studies of wild animals living in the field with all its wonderful complexity and challenges may help redress this balance and re‐kindle interest in this idea among the community from whence it emerged. After all more than 99.999% of all organisms on earth live and age in the field, and not in a protected laboratory, and the processes that cause them to do so may indeed include oxidative stress in one form or another. So studies taking advantage of the huge diversity in aging rate and lifespan in free‐living animals may be particularly relevant and important for gerontologists to be aware of. Finally, free radicals not only play a role in damage, and potentially aging, but are also essential positive components of living systems involved in signaling pathways and in immune function (bactericidal killing). Understanding these functions more clearly may be important in interpreting the confusion of current data. Therefore, meeting people working in these areas to discuss research aims and findings as well may be equally important. However, this cross‐fertilization of disciplines will not happen unless there is a council by which such interactions might be facilitated. There is an urgent need for a forum where ecophysiologists, biogerontologists, and other scientists working on oxidative stress can meet and exchange ideas and data with respect to aging, physiology, ecology, and the evolution of life histories.

## Summary

The role of oxidative stress as a factor influencing aging and longevity, and in driving the evolution of optimal investment patterns between reproduction and survival (life‐history theory) are distinct areas of research with differing underlying theoretical bases. Yet they are interconnected at many different levels. We have formulated a series of questions and propose the collection of some different types of data that will enhance our ability to understand the role of oxidative stress in life histories, that will also impact our understanding of oxidative stress and aging. The six themes highlighted here provide a framework for moving forwards along this alternative path. Significant synergies would be enabled, new insights gained, and much confusion eliminated if there was more dialogue between evolutionary/physiological ecologists and biogerontologists.

## Conflict of Interest

None of the authors has any conflict of interests to declare.
